# Resilience is a dirty word: misunderstood, and how we can truly build it

**DOI:** 10.1186/s13054-022-04040-x

**Published:** 2022-06-08

**Authors:** Mark Z. Y. Tan

**Affiliations:** 1 ST6 Anaesthesia and Intensive Care Medicine, Health Education Northwest, Manchester, UK; 2 National Institute for Health and care Research (NIHR) Academic Clinical Fellow, Humanitarian and Conflict Response Institute, University of Manchester, Manchester, UK

**Keywords:** Resilience, Well-being, Burnout, Organisational culture, Leadership, Mindfulness, Resilience engineering

## Abstract

Resilience is ubiquitous in everyday speech, academic literature and governmental policies. Yet it seems to have taken a narrow scope in healthcare, confined to individual and psychological resilience. This short essay aims to broaden the understanding of resilience to organisational levels and calls intensivists to take active roles in fostering resilience for their staff. The article explores firstly the background and etymology of resilience. It then challenges current approaches and briefly signposts some current work in the area. Some examples of structural factors which build individual resilience are listed, followed by a call for intensivists to take active roles to build future resilience. The need for interdisciplinary, cross-sectoral and multi-level approaches is vital to build future healthcare resilience, and we intensivists must continue to be advocates for systemic change.

*“[Resilience], like pornography, is easier to recognise than it is to define” – Griselda Cooper, Fundamental Principles and Practice of Anaesthesia*

Resilience is a dirty word. It is an overused, poorly understood utterance which appears to consist of a blasphemous, hollow cacophony of yoga, coffee vouchers and mindfulness training. Yet it has crept into all aspects of society, from everyday communications, through academic literature, all the way up to governmental priorities. Despite such ubiquity, it seems to have slowly aggravated the very fragilities it aims to strengthen, evoking eye-rolling, toe-curling, blood-boiling reactions to its use.

We have misunderstood resilience. The Latin “resilire” describes an ability—usually of a material—to “bounce back”, but merely superimposing such a definition onto an individual is akin to considering a single intervention as a panacea. Humans can neither be deemed laboratory conditions, nor our psyche viewed through an elemental lens. Despite this, academic literature tends to focus on acute interventions, on an individual-level, limited to psychological well-being. For example, many studies attempt to determine how best to boost the morale of staff [1]. Or think about the numerous mental health resources now available to us, from National Health Service (NHS) practitioner health to the European Society of Intensive Care Medicine (ESICM) academy’s well-being at work modules. Yet, the terrifying rates of burnout amongst healthcare workers suggest the gross inadequacy of such approaches [2].

We should know better than to apply one-dimensional solutions to multi-faceted problems. Resilience cannot merely be confined to individual interventions. Fortunately, there is slow but increasing recognition of the interdisciplinary, cross-sectoral and multi-level (nicely abbreviated as ICM) approaches required to build true resilience. On an organisational level, resilience has been intricately tied to patient safety paradigms, and its definition spans across a crisis, encompassing the ability to anticipate, respond, monitor and learn from shocks [3]. In that sense, perhaps we should be taking a more organisational perspective of resilience-building, reframing the patient-safety lens to include staff-safety too, and not limiting provisions to the confines of a crisis. The Intensive Care Society is undertaking research to better understand such systemic factors affecting individual well-being and resilience [4]. Universities are also taking notice, with studies on practical factors that build staff resilience. Even the NHS operational strategy for 2022/23 specifically considers how we can adapt to the flexible working needs of the modern healthcare professional [5].

Simple organisational provisions such as clean and functional changing rooms and scrubs can boost resilience, far more than an e-learning module can. Provision of adequate Personal Protective Equipment (PPE) during a pandemic clearly fulfils basic needs far better than coffee-vouchers. Administrative nightmares which plague the start of any rotational job could be streamlined and make working for a new organisation a dream, in a more practical way than any dream-like state achieved during yoga. Processes that enable practitioners to smoothy perform their work cultivates more mindfulness than the stress of needing to improvise workarounds just to provide good patient care. The list goes on. On the face of it, such factors seem insignificant, petty even. Yet, as accumulation of microaggressions can cause lasting psychological damage [6], so an accumulation of such “micro-irritations” may indeed aggravate burnout in a similar fashion.

Let’s not stop at the organisational level (or meso-level in resilience studies). Indices designed for systems level (or macro-level), which had largely been ignored prior to COVID-19, specifically list essential supplies (drugs and equipment for example), and surge capacity as key domains. These include the World Health Organisation’s Building Blocks of Health, the Global Health Security Index (GHSI) and the Epidemic Preparedness Index (EPI). But these seem at constant loggerheads with the normal meso-level priorities of minimising performance variability and maximising cost-effectiveness. These conflicting interests are repeated at the next level, perhaps partially explaining why practitioners often feel unheard by hospital managers. With pressures on both sides, it is little wonder that middle management is literally: caught in the middle. The “redundancies” which are required for surge capacity are instead labelled as “inefficiencies”, generating a negative spin on a potentially positive trajectory. But even these indices could not accurately predict resilience. Many high-scoring countries on the GHSI and EPI performed poorly during the COVID-19 pandemic [7]. The focus on measurement of physical capacities unfortunately pushes the relational aspects of resilience to the side-lines. This in turn deviates from a systems-thinking approach which underpins complex organisations. We must therefore re-frame resilience to prioritise relational aspects in addition to the physical aspects already embedded in resilience-building.

The implementation and advocacy of systemic changes require effective leadership; leadership that considers contrasting priorities, coordinates diverse actors, and operates complex systems to achieve desired goals. Sounds familiar? The reality of critical care medicine makes us intensivists well-suited to be leaders for resilience too. After all, consider the complexity of our daily multi-disciplinary rounds, juggling multiple life support modalities and managing the interdependency of body systems. The dynamicity and responsiveness we have demonstrated as a global ICM community throughout COVID-19 speaks of our ability to respond to a crisis. Our solidarity around ICU data has provided robust and real-time monitoring of the situation. And as we seek to recover and make sense of our experiences over the past two years, we have the opportunity to learn and anticipate the next crisis better. So continue to sound the alarms for staff welfare. Maintain the vigil for those at risk of burnout. Model what we all desire in a supportive culture. We can shift to focus to achieve a better balance between physical and relational aspects of resilience, between staff- and patient-centredness, and between healthcare facilities and the populations they serve.

It is this learning that challenges the etymology of resilience. We must not be satisfied to simply “bounce back” to pre-COVID conditions. Instead, that we have begun to acknowledge the brokenness of the system are inklings of a deeper, more connected understanding of resilient systems, organisations and individuals. By addressing such interactions, we remove the dirt that has limited our vision, and with a clearer view, not merely bounce back, but leap higher, into the future.

*Resilience: from the Latin base “resilire”, to bounce back, from the fundamental base “salire”, to leap.*

## Data Availability

Not applicable.
